# Construction of Thermo-Responsive Elastin-Like Polypeptides (ELPs)-Aggregation-Induced-Emission (AIE) Conjugates for Temperature Sensing

**DOI:** 10.3390/molecules23071725

**Published:** 2018-07-14

**Authors:** Zhe Chen, Zhaoyang Ding, Guangya Zhang, Leilei Tian, Xuanjun Zhang

**Affiliations:** 1 Faculty of Health Sciences, University of Macau, Taipa 999078, Macau, China; yb67616@umac.mo (Z.C.); zyding1@uw.edu (Z.D.); 2 Department of Materials Science and Engineering, Southern University of Science and Technology, Shenzhen 518055, China; 3 Department of Biotechnology and Bioengineering, Huaqiao University, Xiamen 361021, China; zhgyghh@hqu.edu.cn

**Keywords:** elastin-like polypeptides (ELPs), aggregation-induced-emission (AIE), thermo-responsive, temperature sensing

## Abstract

In this work, an aggregation-induced emission (AIE) molecule (tetraphenylethene derivative, TPE-COOH) was conjugated to elastin-like polypeptides (ELPs40) via an amide bond to form ELPs40-TPE. The successful synthesis of ELPs40-TPE was confirmed by Circular Dichroism spectroscopy, gel electrophoresis, UV-vis absorption, and fluorescence emission spectroscopy. ELPs40-TPE possessed both amphiphilicity and the features of an AIE, and the fluorescence intensity was dependent on the local temperature. The Hela cells imaging indicated that ELPs40-TPE has great potential for bio-imaging applications because of its advantages of high fluorescence intensity, good water-solubility, and remarkable biocompatibility.

## 1. Introduction

Stimuli-responsive materials are becoming increasingly attractive in biomedical applications [1]. Such materials are characterized by a change in properties/behavior upon external stimuli [2], including light [3], temperature [4,5], electric field [6], and pH [7]. Among them, thermo-responsive materials have huge potential in biomedical applications due to their capability of phase transition above or below a certain temperature [8,9,10]. 

Elastin-like polypeptides (ELPs) are candidates with thermo-responsive characteristics for in vivo applications [11]. ELPs consist of Val-Pro-Gly-Xaa-Gly (VPGXG) pentapeptide repeats, where X represents the guest residue (any amino acid other than proline). ELPs undergo a reversible phase transition at their inverse transition temperature (T_t_) [12]. Below the T_t_, ELPs disperse well and exist as disordered structures in water; while above the T_t_, ELPs form aggregates. The T_t_ of ELPs is highly tunable by altering molecular parameters, such as amino acid composition and molecular weight, and the T_t_ can be accurately adjusted between 0 °C and 100 °C, which can be applied to a wider area of temperature-sensitive applications [13]. Moreover, the biological composition of ELPs ensures its low cytotoxicity, good biocompatibility, and biodegradation [14]. Therefore, ELPs are extensively applied in vivo for their unique stability and drug activities [15,16], thus investigation of the thermal responsive property of ELPs is significant.

However, there are few studies that report the temperature response of ELPs through fluorescence methodology. The thermal transition of ELPs can be monitored by conjugating fluorescent molecules, where the fluorophores must have the characteristics of high sensitivity and fast response [17]. Conventionally used fluorogens usually suffer from the aggregation-caused quenching (ACQ) effect that is caused by π–π stacking interactions, which restricts their applications when they are used at a high concentration [18]. In 2001, Tang’s group [19] discovered an extraordinary photophysical phenomenon opposite to ACQ where luminogens become non-emissive in good solvents and intensively emit as solid aggregates in poor solvents. They termed the phenomenon aggregation-induced emission (AIE), and the fluorophores with the AIE property were termed as AIEgens. Thus the advantages of strong sensitivity, high selectivity, fast response, and low background noise make AIEgens mostly used as turn-on fluorescent probes for bio-sensors [20,21,22] and bio-imaging [23,24,25]. Inspired by the great fluorescence performance of AIEgens, we designed a new thermo-sensitive material by incorporating an AIE molecule into ELPs to explore its temperature-responsive behavior and cell imaging potential.

In this work, we used ELPs [KV8F-40] (abbreviated as ELPs40) which contained a sequence of 40 (VPGXG) pentapeptide repeats, the fourth amino acid residue of the repeats consisted of Lys, Val, and Phe in a 1:8:1 ratio. A typical AIE molecule 4-((4-(1,2,2-Triphenylvinyl) benzyl) oxy) benzoic acid (abbreviated as TPE-COOH) was used as the fluorescent marker. It was hypothesized that after the hydrophobic TPE-COOH was conjugated to hydrophilic ELPs40 via amidation, the resulting ELPs40-TPE conjugates could exhibit both water solubility and temperature sensitivity. It was the first example of an ELPs-based amphiphilic material with AIE properties. The transformation of the conformation of ELPs40-TPE occurred as the temperature changed, which led to a difference in its fluorescence. We anticipated that the novel material would show the capacities of temperature-responsive fluorescent variation and excellent biocompatibility and be used as a fluorescent probe for cell imaging.

## 2. Results and Discussion

### 2.1. Synthesis of ELP40-TPE

**Scheme 1:**
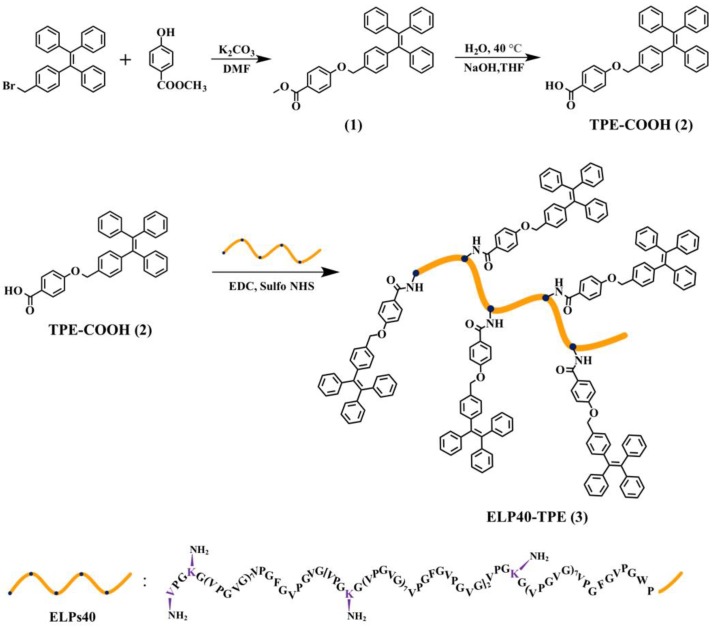
Synthetic route of ELPs40-TPE.

ELPs40-TPE was synthesized via an amidation reaction between the carboxyl-functionalized TPE derivative (TPE-COOH) and the amine-containing ELPs40 peptide as shown in Scheme 1, in which EDC and sulfo-NHS were used to activate the carboxyl group for efficient coupling. The synthetic processes of the representative AIE molecule TPE-COOH (**2**) was based on the reported literature [26] (all the compounds were characterized by ^1^H NMR, ^13^C NMR spectroscopies (Figures S1–S4). The activation reaction of the carboxyl group of (**2**) was carried out in DMSO, while the conjugation reaction of ELPs40 and (**2**) took place in a DMSO–water mixture. The resultant ELPs40-TPE was purified through dialysis and ultrafiltration. From the composition of the amino acid sequence of ELPs40 (Table 1), there was one amine at the N-terminus and four amines at the lysine residues, thus up to five TPE molecules could be connected to each ELPs40 chain. The TPE:ELPs40 average molar conjugation ratio was about 2.5, which was determined by the UV-vis absorbance spectra of ELPs40-TPE (according to the Equation (1), see the *Materials and Methods* for calculational details). Considering the steric hindrance, the resulting label ratio was reasonable.

### 2.2. Characterizations of ELPs40-TPE

**Figure 1:**
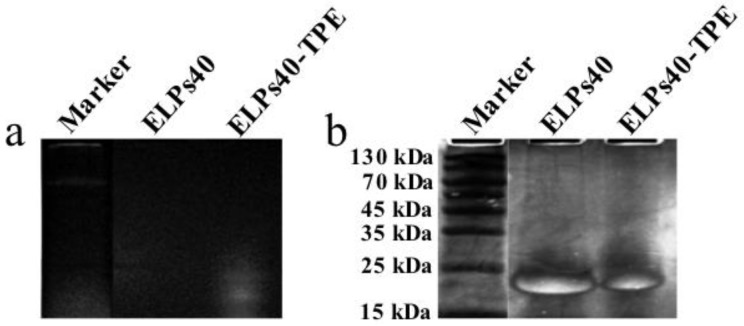
12.0% SDS-PAGE analysis of ELPs40 and ELPs40-TPE. The gel was visualized under UV lamp first (**a**), and then stained with 0.5 M CuCl_2_ (**b**).

**Figure 2:**
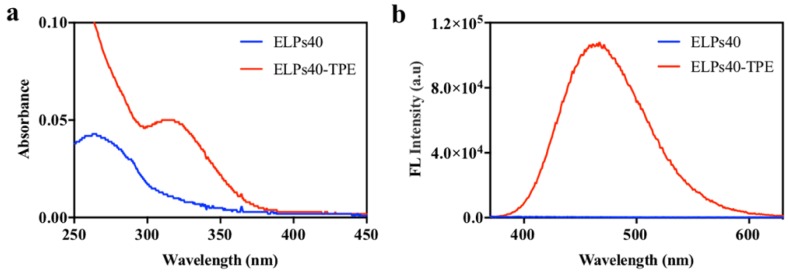
(**a**) UV-Vis absorption spectra of ELPs40 (blue) and ELPs40-TPE (red) in aqueous solution. (**b**) Fluorescence emission spectra of ELPs40 (blue) and ELPs40-TPE (red) in aqueous solution. The concentrations of ELPs40 and ELPs40-TPE were 5 µM (ELPs40 concentration was determined using the protein absorbance at 280 nm and the protein’s molar extinction coefficient (7190 L/mol cm) [31]), λ_ex_ = 320 nm.

The ELPs40-TPE was characterized by Circular Dichroism (CD) spectra (Figure S5), gel electrophoresis (Figure 1), UV-Vis absorption spectra (Figure 2a), and fluorescence emission spectra (Figure 2b). CD spectroscopy was usually used for the characterization of ELPs40 peptide by its unique secondary structure, where a positive peak in the region of 206 nm to 212 nm was characteristic of a type II β-turn [27,28]. As shown in Figure S5, in the CD spectra of ELPs40 and ELPs40-TPE, negative peaks at about 195 nm and 224 nm and positive peak at about 210 nm correspond to the random coil, helix, and type II β-turn secondary structure of ELPs40, respectively. The accurate composition of the secondary structure of ELPs40 and ELPs40-TPE were calculated through the Pro-Data Viewer program (Table 2). The type II β-turn ratio in the secondary structure of ELPs40-TPE was shown to decrease from 22.2% to 20.4% by conjugating the AIEgen to the ELPs40 peptide. These results indicated that the TPE-COOH molecule underwent interactions with ELPs40 and the synthesis of ELPs40-TPE was successful.

Successful conjugation of TPE-COOH to ELPs40 was further proved by 12.0% sodium dodecyl sulfate-polyacrylamide gel electrophoresis (SDS-PAGE) as shown in Figure 1. The gel was directly visualized under UV lamp to observe the fluorescence of TPE-containing band in the lane of ELPs40-TPE (Figure 1a), which was ascribed to the aggregation of the AIE groups in ELPs40-TPE. After further staining of the same gel with CuCl_2_ for 2h (Figure 1b), the ELPs40-TPE lane displayed the same band due to ELPs40. Besides, the ELPs40 lane showed a clear band at the same location (between 15 kDa and 25 kDa, where the molecular weight of ELPs40 was about 17 kDa). Under the UV light, the smearing of the ELPs40-TPE band in the gel indicated that ELPs40 was decorated with TPE but with a diverse label degree, which resulted in different electrophoretic migration rate and the appearance of smearing. 

Then, the optical properties of ELPs40-TPE and ELPs40 were studied by measuring their absorption and emission spectra at the same concentration. From Figure 2a, both ELPs40 and ELPs40-TPE showed the absorption maxima at about 270 nm, attributed to the peptide structure, while ELPs40-TPE exhibited another absorption peak at about 320 nm due to the TPE moiety. As we expected, the ELPs40 peptide endowed ELPs40-TPE with good water solubility. However, ELPs40-TPE emitted a strong fluorescence in water (Figure 2b). This interesting phenomenon different from the conventional AIE effect, where AIEgens usually exhibited non-emissive in good solvents. The conjugation of AIEgens with ELPs40 enhanced the hydrophobicity of ELPs40, thus the amphiphilicity of ELPs40-TPE conjugates made the TPE moiety aggregate and emit intense fluorescence, which was similar to the hydrophobic small molecule conjugated ELPs-dox system [29,30]. 

### 2.3. AIE Property of ELPs40-TPE

**Figure 3:**
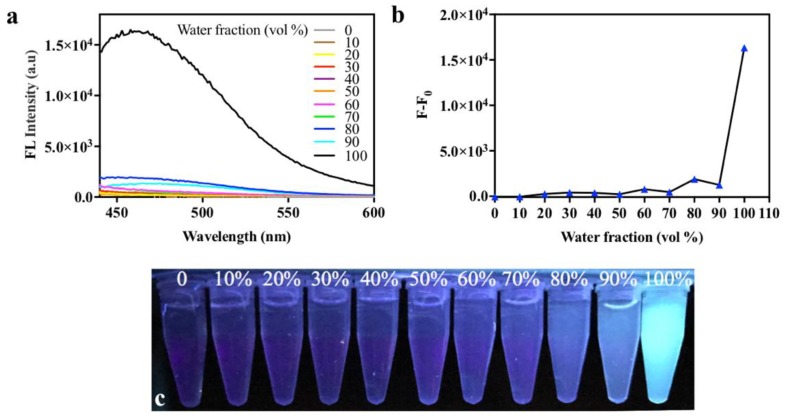
(**a**) Fluorescent spectra of ELPs40-TPE in the DMF–water system with different water fractions. (The concentrations ELPs40-TPE was 5 µM, λ_ex_ = 320 nm); (**b**) The maximum fluorescence intensity of ELPs40-TPE in the DMF–water system at 460 nm. (F_0_ is the fluorescence intensity of blank background, the concentrations of ELPs40 and ELPs40-TPE were 1 µM, λ_ex_ = 320 nm); (**c**) Photographs of ELPs40-TPE under UV light (365 nm) in different water fractions.

In order to elucidate the phenomenon of strong fluorescence of ELPs40-TPE in water, the AIE effect of ELPs40-TPE was investigated by fluorescent spectra in DMF/water system with different water fractions. From Figure 3, ELPs40-TPE showed a weak fluorescence, because it was completely dispersed in pure DMF and low water fractions (f_W_ < 50%). However, when the fraction of water increased to 90%, ELPs40-TPE suddenly emitted remarkable fluorescence. It is well known that the mechanism of AIE is based on mainly Restriction of Intramolecular Motions (RIM), which includes Restriction of Intramolecular Rotations (RIR) and Restriction of Intramolecular Vibrations (RIV) [20]. In the aggregated state, the restriction of such motions resulted in the blocking of the non-radiative pathway, which made the AIEgens emit strong fluorescence [32]. Thus, the aggregation was regarded as a necessary requirement [33], while Kazuki and coworkers recently pointed out that aggregation was a sufficient condition for AIE rather than a necessary requirement [34]. ELPs40-TPE existed as a random coil conformation in aqueous solution and the hydrogen bonds formed between acid amides groups and water molecules increased the rigidity of the environment. Besides, the hydrophobic effect effectively promoted the aggregation of the hydrophobic AIE-featured TPE group of the ELPs40-TPE chain. Therefore, both the rigidity-enhanced environment and hydrophobic effect restricted the intermolecular motions and inhibited non-radiative pathway, leading to strong emission. In contrast, ELPs40-TPE was non-emissive in pure DMF, owing to the free intramolecular motions of phenyl rings in TPE segments and the flexibility of ELPs40 peptide chain. 

### 2.4. Temperature Responsivity of ELPs40-TPE

**Figure 4:**
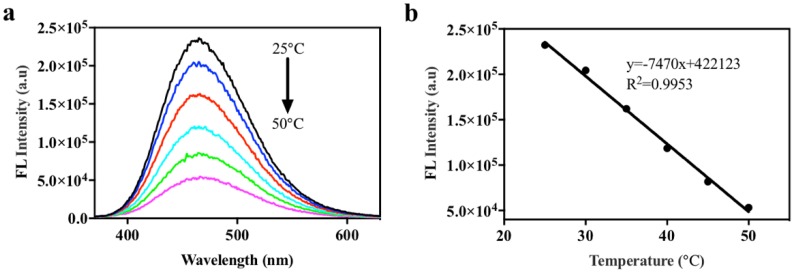
(**a**) The fluorescent spectra of ELPs40-TPE in water at different temperatures. (**b**) Linear relationships between the fluorescence intensity of ELPs40-TPE at 460 nm and temperature from 25 °C to 50 °C. The concentration of ELPs40-TPE was 10 µM, λ_ex_ = 320 nm.

**Scheme 2:**
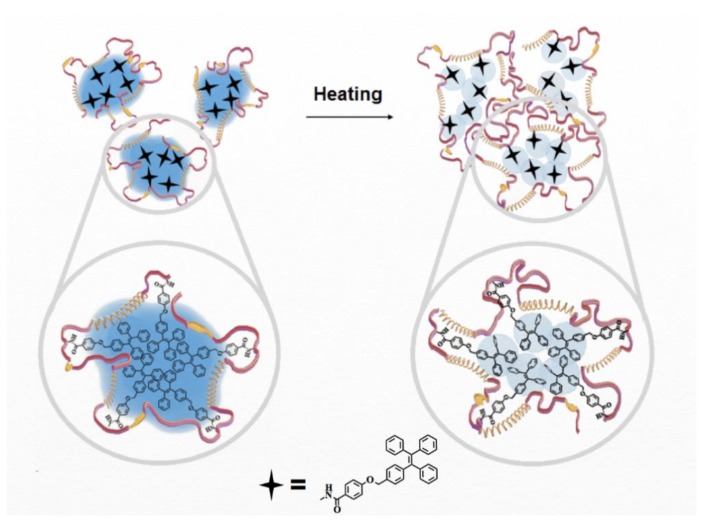
Mechanism for temperature-responsive behavior of ELPs40-TPE in aqueous solution.

We then explored the temperature responsiveness of ELPs40-TPE in water. As temperature increased from 25 °C to 50 °C in steps of 5 °C, the fluorescent intensity of ELPs40-TPE decreased gradually (Figure 4). In the range of 25 °C to 50 °C, the plot of fluorescence intensity at 460 nm of ELPs40-TPE as a function of temperature was in a good linear regression with a correlation coefficient of 0.9953. The T_t_ of ELPs was related to its sequence, chain length, polypeptide concentration, and the type and concentration of solution [12]. According to the reported literature [35], the T_t_ of ELPs40 was more than 53 °C in water, which was consistent with the phenomena during the process of heating (the solution of the ELPs40-TPE was always transparent). The mechanism of temperature-responsive behavior of ELPs40-TPE in aqueous solution was shown in Scheme 2. The hydrophobic effect made the TPE groups of ELPs40-TPE aggregate and emit a strong fluorescence at 25 °C. Raising the temperature resulted in two different effects on ELPs40-TPE. As the temperature rose, the ELPs40 peptide of ELPs40-TPE gradually accumulated, which synchronously accelerated the intramolecular rotation of TPE moieties and the motion of whole conjugates. The former effect enhanced the emission of the TPE groups, and the latter effect weakened the emission. ELPs40-TPE had already aggregated in aqueous solution at 25 °C, thus the latter effect played the dominant role during the heating, and the fluorescence intensity gradually decreased.

**Figure 5:**
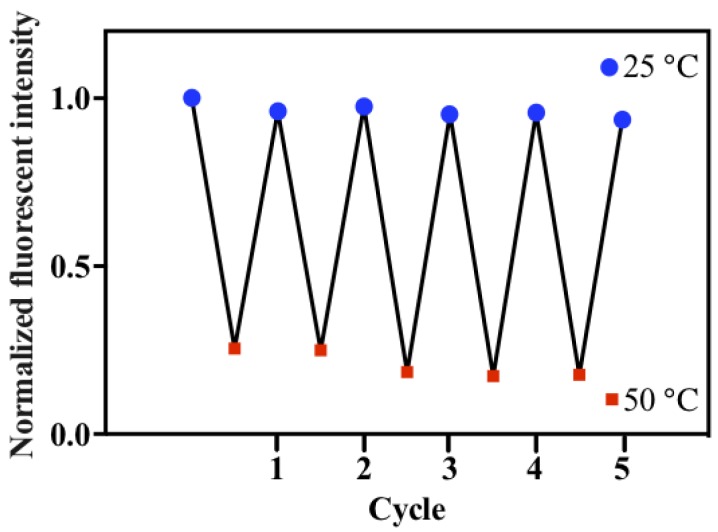
The reversibility of ELPs40-TPE. The concentration of ELPs40-TPE was 5 µM, λ_ex_ = 320 nm.

The reversibility of the temperature responsiveness of ELPs40-TPE was then investigated by heating the aqueous solution of ELPs40-TPE to 50 °C and then cooling it to 25 °C. As shown in Figure 5, the fluorescence intensity of ELPs40-TPE gradually increased as the temperature returned to 25 °C. During the reduction of temperature, there were also two effects to the ELPs40-TPE. The ELPs40 peptide chain gradually disaggregated, which made the TPE fluorogen emit weaker fluorescence, while the intramolecular rotations of TPE moiety was hindered with the reduction in temperature, which caused stronger fluorescence. Ultimately, the latter effect acted as the major role during the cooling process. The results of the heating–cooling cycle (25 °C–50 °C) of ELPs40-TPE indicated its reversibility. 

### 2.5. DLS Study

**Figure 6:**
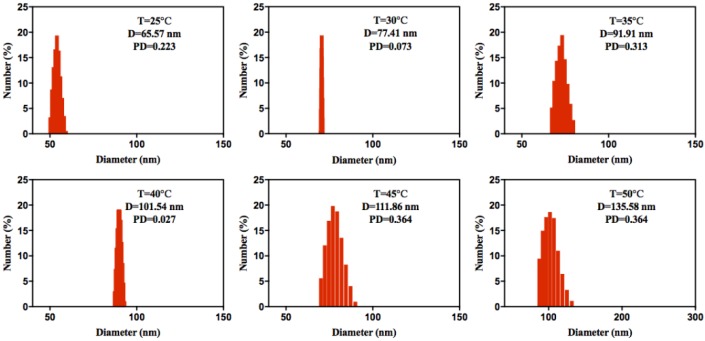
Particle size distributions of ELPs40-TPE in aqueous solution under different temperatures.

DLS was used to monitor the temperature response of ELPs40-TPE in aqueous solution. As shown in Figure 6, the results displayed that the ELPs40-TPE conjugates indeed aggregated at 25 °C, and the size of ELPs40-TPE increased from 65 nm to 135 nm in the temperature range of 25 °C to 50 °C. The DLS outcomes of ELPs40-TPE further demonstrated the mechanism of temperature-responsive behavior we proposed in *2.4. Temperature Responsivity of ELPs40-TPE*.

### 2.6. Cell Imaging Applications

**Figure 7:**
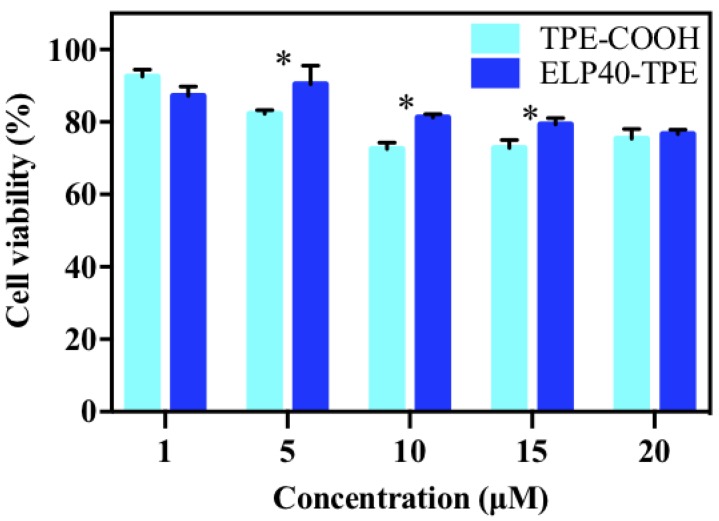
Cell viability (%) of Hela cells treated with different concentrations of ELPs40-TPE and TPE-COOH for 24 h by MTT assay. Data as mean values ± SD (*n* = 3). The Student’s *t*-test was used to compare the results of the experiments. A * *p*-value < 0.05 was considered significant.

Based on the amino acid components of the ELPs40 peptide chain, it was deduced that ELPs40-TPE might display good biocompatibility. MTT cell viability assays were conducted to evaluate the cytotoxicity of ELPs40-TPE. Hela cells were incubated with different concentrations of ELPs40-TPE at 37 °C for 24 h, while TPE-COOH was used as the control (Figure 7). As shown in Figure 7, ELPs40-TPE exhibited lower cytotoxicity and better biocompatibility than TPE-COOH in the range of 5–15 µM. However, they showed comparable biocompatibility across all concentrations. 

**Figure 8:**
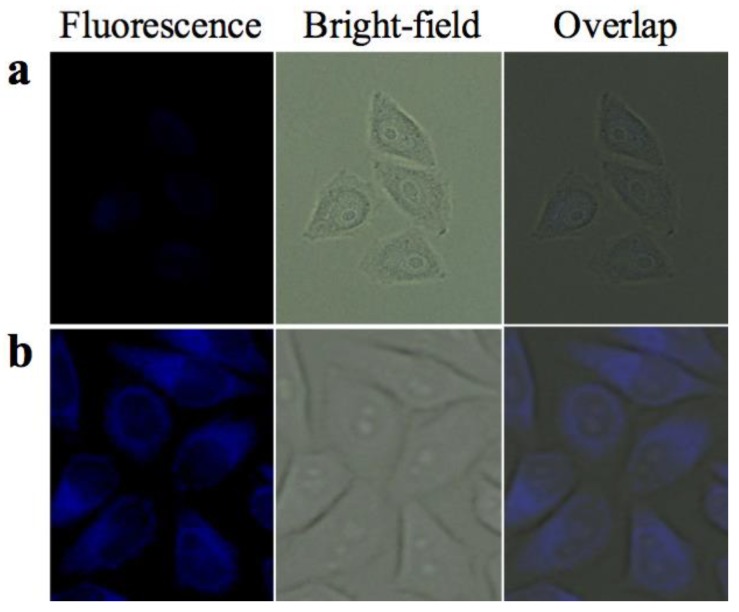
Fluorescent images of HeLa cells stained with (**a**) TPE-COOH for 30 min and (**b**) ELPs40-TPE conjugates for 30 min. λ_ex_ = 365 nm. (The concentration of TPE was identical, 10 µM).

The AIE property and the results of cell viability suggested that ELPs40-TPE might be utilized as a fluorescent bioprobe for cell imaging (Figure 8). The uptake of small particles (less than 200 nm) by cells was generally through the endocytosis pathway [36]. As shown in Figure 8, the cytoplasm of Hela cells emitted a strong blue fluorescence, demonstrating that the ELPs40-TPE probe entered into the cytoplasm in the aggregated form. In contrast, Hela cells stained by TPE-COOH only exhibited a weak fluorescence, probably due to the low uptake efficiency of TPE-COOH. The hydrophobic TPE-COOH was prone to aggregation in cell culture medium and could be subsequently cleared by phosphate buffer. Thus, the amphiphilic ELPs40-TPE fluorescent probe with AIE characteristics showed promising biocompatibility for bio-imaging.

## 3. Materials and Methods

### 3.1. Materials

1-[(4-bromomethyl)-phenyl]-1,2,2-triphenylethene and 4-methyl hydroxybenzoate were purchased from Rhea Biotech Company (Xian, China). Potassium carbonate, anhydrous MgSO_4_, sodium hydroxide, tetrabutylammonium bromide (TBAB), 1-ethyl-3-(-3-dimethylaminopropyl) carbodiimide hydrochloride (EDC), and N-hydroxysulfosuccinimide sodium salt (sulfo-NHS) were purchased from Sigma-Aldrich. Dialysis bag (3 KDa) was purchased from Sangon Biotech Company (Shanghai, China), Ultra-4 Centrifugal Filter Devices (3 KDa) was purchased from Merck Millipore (Darmstadt, Germany). All the solvents of analytical grade were employed without more purification. ELPs40 was kindly provided by Guangya Zhang’s Group (Huaqiao university).

### 3.2. Synthesis of Methyl 4-((4-(1,2,2-triphenylvinyl) benzyl) oxy) Benzoate (**1**)

1-[(4-bromomethyl)-phenyl]-1,2,2-triphenylethene (0.427 g, 1 mmol), 4-methyl hydroxybenzoate (0.42 g, 2 mmol), and potassium carbonate (0.553 g, 4 mmol) were mixed in of DMF (20 mL), the mixture was heated to 80 °C overnight. After the reaction was completed, the mixture was poured into ice water and filtered, and the solid product was then dissolved in CH_2_Cl_2_ and washed with 1 M NaOH (50 mL × 3) and dried over anhydrous MgSO_4_. After the evaporation of solvent, the crude product was purified by a silica gel column using CH_2_Cl_2_/hexane (*v*/*v* 1:1) as eluent to give compound **(1)** as a white solid (0.195 g, 40% yield). ^1^H NMR (400MHz, CDCl_3_), δ (TMS, ppm): 8.01 (d, 2H, ArH), 7.19 (s,1H, ArH), 7.18 (s, 1H, ArH), 7.11–7.14 (m, 9H, ArH), 7.09 (s, 1H, ArH), 7.03–7.07 (m, 7H, ArH), 6.98 (d, 2H, ArH), 5.04 (s, 2H, -ArCH2-), 3.92 (s, 3H, -COOCH3). ^13^C NMR (100 MHz, CDCl_3_) δ: 166.9, 162.5, 143.6, 141.4, 140.4, 134.2, 131.6, 131.3, 127.7, 127.7, 126.8, 126.5, 122.8, 114.5, 70.0, 51.9.

### 3.3. Synthesis of 4-((4-(1,2,2-triphenylvinyl) benzyl) oxy) Benzoic acid (**2**)

The THF solution of compound **(1)** (100 mg/10 mL) was added to 10 mL 30% NaOH solution, then 30 mg tetrabutylammonium bromide (TBAB) was mixed to the mixture and stirred at 70 °C overnight and cooled to room temperature. The reaction mixture was then neutralized with 1 M HCl until the white precipitate appeared (pH was about 7) followed by the addition of ethyl acetate for extraction. The white solids were obtained after the evaporation of solvent (70 mg, 70% yield). ^1^H NMR (400 MHz, DMSO-d6), δ (TMS, ppm): 7.88 (d, 2H, ArH), 7.21 (d, 2H, ArH), 7.12–7.17 (d, 9H, ArH), 7.06 (d, 2H, ArH), 6.95–7.02 (m, 8H, ArH), 5.07 (s, 2H, -ArCH2-). ^13^C NMR (100 MHz, DMSO-d6) δ: 167.6, 162.3, 143.6, 143.4, 141.3, 140.7, 135.1, 131.8, 131.2, 131.1, 128.3, 127.9, 127.1, 115.0, 69.8.

### 3.4. Synthesis and Purification of ELPs40-TPE (**3**)

0.4 mg 1-ethyl-3-(-3-dimethylaminopropyl) carbodiimide hydrochloride (EDC) and 0.6 mg N-hydroxysulfosuccinimide sodium salt (sulfo-NHS) were added to a solution of (**2**) in DMSO (1 mL, 2 mg/mL). The solution was shaken slightly for 1 h on a 37 °C constant thermo-shaker. Then 1 mL ELPs40 solution (1 µmol) was added to the reaction mixture and continued shaking for 2 h.

The ELPs40-TPE reaction solution was transferred to a 3 K dialysis bag for dialysis for 48 h. After dialysis, the crude products in the dialysis bag were ultrafiltered via 3 K ultra-4 centrifugal filter devices, and 400 µL ELPs40-TPE concentrate was collected. The TPE concentration was quantified by the calibration curve drawn from the UV absorption of TPE-COOH at 320 nm. The ELPs40 concentration was calculated using the derived Equation (1) [31]:

(1) $$[\mathrm{ELPs}40]\;=\;\frac{\left(\mathrm{Abs}280\;\mathrm{nm}\;-\;(1.063\;\times \;\mathrm{Abs}320\;\mathrm{nm})\right)}{\varepsilon \;\times \;1\;\mathrm{cm}}\;$$

where ε was the molar extinction coefficient of the protein (ε = 7190 L/mol cm). The ELPs40-TPE concentrates was stored in −20 °C refrigerator for subsequent analysis.

### 3.5. Characterizations

#### 3.5.1. Circular Dichroism (CD) Spectroscopy Measurements

Circular Dichroism (CD) spectra were studied on a Chirascan-plus Circular Dichroism Spectrometer (Applied Photophysics Ltd., Leatherhead, Surrey, UK). Measurements were conducted in a quartz cell with a 2 mm path length over the range of 190–300 nm in the nitrogen atmosphere. The concentrations of ELPs40-TPE and ELPs40 were 10 µM in aqueous solution.

#### 3.5.2. Gel Electrophoresis Analysis

Gel electrophoresis was performed using 12.0% SDS-PAGE at 120 V for 1 h. The gel images were captured on gel image system (Bio-Rad, Hercules, CA, USA).

#### 3.5.3. UV-Vis Absorbance Spectroscopy and Fluorescence Emission Spectroscopy Measurements

UV-vis absorbance spectra were measured via a UV-1800 spectrophotometer (SHIMADZU, Japan) and a quartz cell with 1 cm optical pathway. Fluorescence emission spectra were measured with a Fluoromax-4 spectrofluorometer (HORIBA, Edison, NJ, USA) and a quartz fluorescence cuvette (2 mm × 10 mm), and with the following settings: λ_ex_ = 320 nm, λ_em_ = 460 nm.

#### 3.5.4. DLS Measurements

Dynamic light scattering (DLS) measurements were performed on Brookhaven NanoBrook Omni instrument (Brookhaven Instruments Corporation, New York, NY, USA). The concentration of ELPs40-TPE was 5 µM in aqueous solution.

#### 3.5.5. Cell Culture

HeLa cells were provided by the Faculty of Health Sciences, University of Macau, which were cultured in RPMI-1640 medium containing 10% Fetal Bovine Serum (FBS) and 1% penicillin–streptomycin in a humidity incubator (5% CO_2_, 37 °C). 

#### 3.5.6. Cell Imaging

The cytotoxicity assay was performed before cell imaging by using a methyl thiazolyl tetrazolium (MTT) Cell Proliferation and Cytotoxicity Assay Kit (Beyotime, Shanghai, China) against HeLa cells. The absorbance of MTT at 570 nm was recorded by the Tecan plate reader (Tecan, Männedorf, Switzerland). Cell viability was estimated by the ratio of the absorbance of the cells incubated with ELPs40-TPE to that of the cells incubated with RPMI-1640 medium only. HeLa cells were precultured on 35 mm glass bottom cell culture dishes in incubator. After 80% confluence, the cells were washed three times with 1 × PBS buffer. Then the cells were incubated with ELPs40-TPE and TPE-COOH for 30 min at 37 °C, respectively (the concentration of TPE was identical, 10 µM). After the incubation, the cells were washed three times with 1 × PBS buffer. Fixative solution (Histochoice^®^ Mb Tissue Fixative, Amresco, Solon, OH, USA) was added at room temperature for 15 min, then the cells were washed with 1 × PBS buffer and used for cell imaging.

## 4. Conclusions

We have synthesized a novel amphiphilic ELPs40-TPE material by using hydrophilic ELPs40 peptide and hydrophobic TPE-COOH molecule via amidation. The obtained ELPs40-TPE conjugates with AIE attributes were different from the conventional AIE fluorogens, which exhibited strong fluorescence in aqueous solution and good water solubility. Moreover, the ELPs40-TPE conjugates were temperature-responsive and their fluorescence could be tuned by changing temperature. HeLa cells imaging demonstrated that not only does ELPs40-TPE have great luminescent properties, but also low cytotoxicity and excellent biocompatibility. Thus, we believe that the new material described here could attract much attention because of the above advantages, especially in bio-imaging applications.

## Figures and Tables

**Table 1:** Protein sequence of ELPs40.

	Amino Acid Sequence *
ELPs40	VPGKGVPGVGVPGVGVPGVGVPGVGVPGVGVPGVGVPGVGVPGFGVPGVG
	VPGKGVPGVGVPGVGVPGVGVPGVGVPGVGVPGVGVPGVGVPGFGVPGVG
	VPGKGVPGVGVPGVGVPGVGVPGVGVPGVGVPGVGVPGVGVPGFGVPGVG
	VPGKGVPGVGVPGVGVPGVGVPGVGVPGVGVPGVGVPGVGVPGFGVPGWP

* The underlined amino acids indicate the position of the amines at the N-terminus and the lysine residues. There was a tryptophan codon added to the end of ELPs40 gene to synthesize ELPs40, thus each ELPs40 contained one tryptophan (W).

**Table 2:** The composition of the secondary structure of ELPs40 and ELPs40-TPE.

The Type of Structure	ELPs40	ELPs40-TPE
Helix	18.7%	21.2%
Antiparallel	42.7%	29.6%
Parallel	8.4%	9.0%
Beta-turn	22.2%	20.4%
Random coil	17.7%	23.2%

## Supplementary Materials

The following are available online. Figure S1: ^1^H NMR spectrum of **(1)** in CDCl_3_, Figure S2: ^13^C NMR spectrum of **(1)** in CDCl_3_, Figure S3: ^1^H NMR spectrum of **(2)** in DMSO-d6, Figure S4: ^13^C NMR spectrum of **(2)** in DMSO-d6, Figure S5: CD spectra of ELPs40 and ELPs40-TPE.
