# Supplementary material for: Construction of Thermo-Responsive Elastin-Like Polypeptides (ELPs)-Aggregation-Induced-Emission (AIE) Conjugates for Temperature Sensing
Source: Molecules. 2018 Jul 14;23(7):1725. doi: 10.3390/molecules23071725 (PMC6100176; doi:10.3390/molecules23071725)
Supplement: Supplementary file 1 [file molecules-23-01725-s001.pdf]

Construction of thermo-responsive elastin-like polypeptides (ELPs)-aggregation-induced-emission (AIE) conjugates for temperature sensing

Zhe Chen[1,2], Zhaoyang Ding[1], Guangya Zhang[3], Leilei Tian[2,*] and Xuanjun Zhang[1,*]

[1] Faculty of Health Sciences, University of Macau, Taipa 999078, Macau, China; yb67616@umac.mo (Z.C.); zydingl@uw.edu (Z.D.)

[2] Department of Materials Science and Engineering, Southern University of Science and Technology, Shenzhen 518055, Guangdong, China

[3] Department of Biotechnology and Bioengineering, Huaqiao University, Xiamen 361021, Fujian, China; zhgyghh@hqu.edu.cn (G.Z.)

* Correspondence: tianll@sustc.edu.cn (L.T.); xuanjunzhang@umac.mo (X.Z.); Tel.: +86-0755-88018524 (L.T.); +853-8822-4928 (X.Z.)

1. ^{1}H NMR spectrum of compound (1)

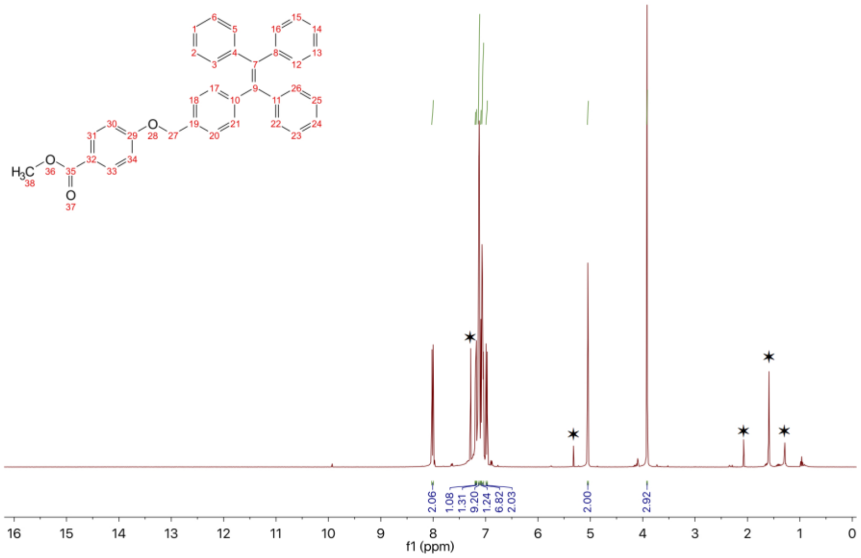

Figure S1. ^{1}H NMR spectrum of **(1)** in CDCl3. The residual solvent signals are marked with asterisks.

2. ^{13}C NMR spectrum of compound (1)

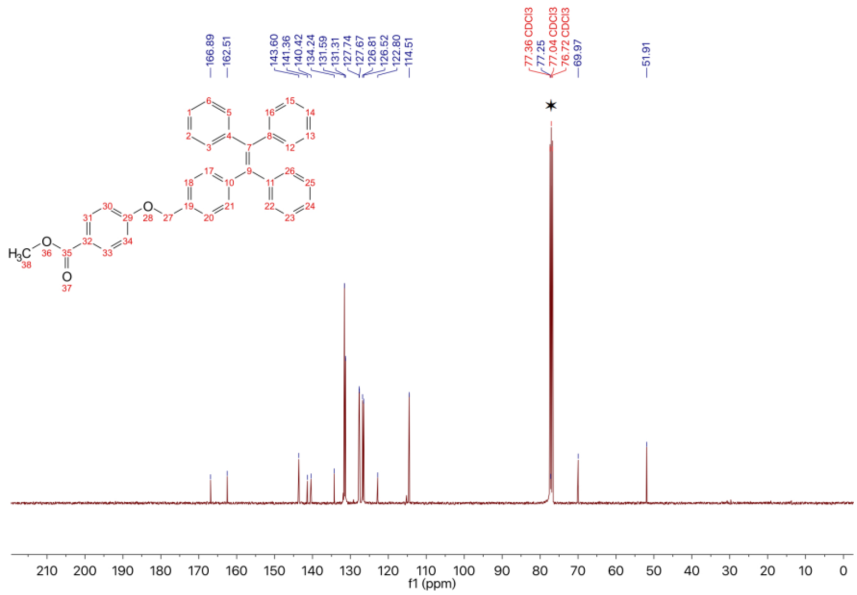

Figure S2. ^{13}C NMR spectrum of **(1)** in CDCl3. The residual solvent signals are marked with asterisks.

3. ^{1}H NMR spectrum of compound (2)

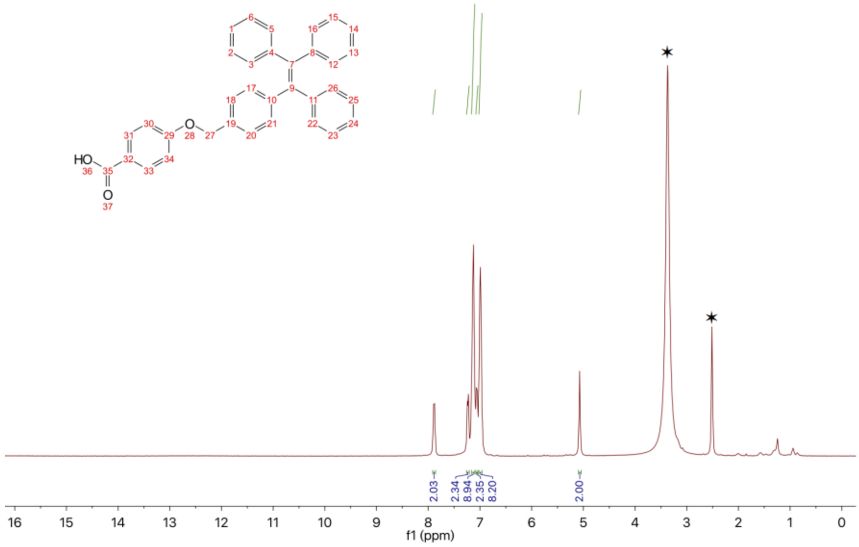

Figure S3. ^{1}H NMR spectrum of **(2)** in DMSO-d6. The residual solvent signals are marked with asterisks.

4. ^{13}C NMR spectrum of compound (2)

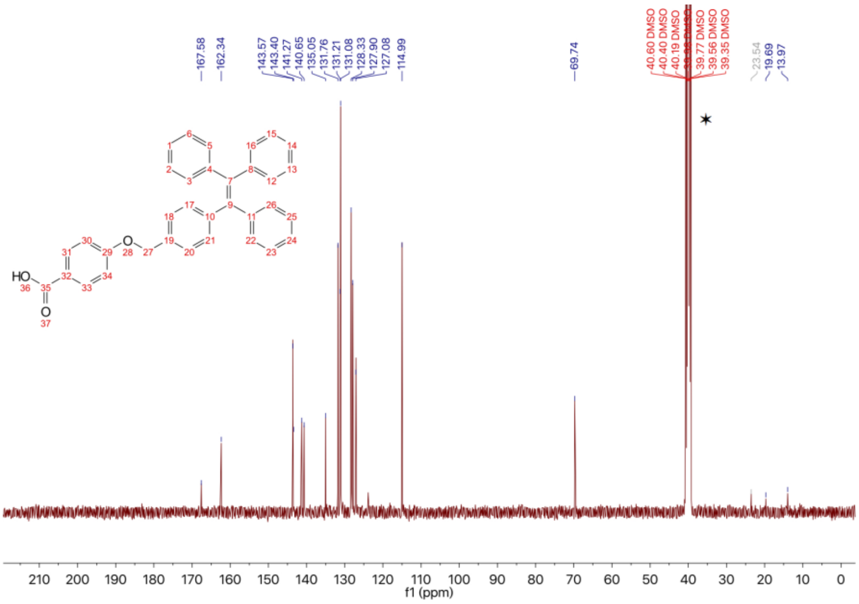

Figure S4. ^{13}C NMR spectrum of **(2)** in DMSO-d6. The residual solvent signals are marked with asterisks.

5. CD spectra of ELPs40 and ELPs40-TPE

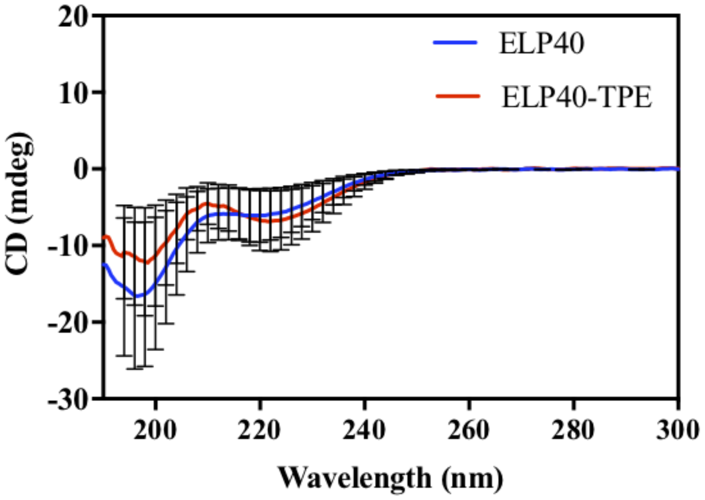

Figure S5. CD spectra of **ELPs40** (blue) and **ELPs40-TPE** (red). The concentrations of **ELPs40** and **ELPs40-TPE** were 10 μM. Data as mean values ± SD (n = 3).
